# Philadelphia Hospital

**Published:** 1844-03-09

**Authors:** 


### JEFFERSON MEDICAL COLLEGE.

### PHILADELPHIA HOSPITAL.

### CLINIC OF THE JEFFERSON MEDICAL COLLEGE.

*February* 3, 1844.

### BY PROFESSOR DUNGLISON.

[Reported by E. R. Squibb, and Samuel G. White.]

#### HEMIPLEGIA.

The attention of the class was first directed to two cases of hemiplegia, which had, on a former occasion, excited their interest. They were presented now merely to exhibit the existing condition, and to report their progress. One is improving gradually, although apprehensions of a second attack must necessarily be entertained. The other, the case of ramollissement, which was referred to briefly at the last clinic, in which the optic nerves or lobes are implicated, has had a second attack, as was prognosticated. There is now a more perfect loss of motion on one side, with a marked affection of the organs of speech, so that she has great difficulty in articulating intelligibly. From this latter phenomenon some pathologists would refer the fresh sanguineous effusion to the anterior lobes of the cerebrum, to which the encephalic organ of language is referred by the phrenologists. The propriety of this location is not, however, established.

In connection with this subject the lecturer remarked, that not unfrequently he thought an error was committed in having recourse to bleeding too early in cerebral hemorrhage. A shock is given at the time, and he thought it was always better, as in concussion of the brain, to wait until the effects of the shock, the feebleness of circulation, and paleness, have passed away. The objects, with which bloodletting is practised, are either to prevent further effusion where evidences of activity of circulation exist, or to aid in the absorption of the blood that has been already extravasated.

### EPILEPSY.

The Professor then proceeded to make some observations on epilepsy; and remarked, that as we have organic and functional apoplexy, so may we have organic and functional epilepsy; still, as he had observed in a former lecture, in regard to certain neuroses, the neurine, it is presumable, must always be altered in this affection also; yet inasmuch as there are no appearances on dissection, which can be regarded as pathognomonic, epilepsy has properly been classed amongst the neuroses, according to the definition usually given of the term.

Whatever may be the nature of the modification of the neurine, it gives rise to phenomena that are well marked, as loss of sensation, volition, and mental and moral manifestation. It is distinguished from apoplexy by the absence of stertorous breathing, and by the accompanying convulsions. The paroxysms occur oftener during the night than the day, and this has by some been supposed to be attributable to the greater quantity of blood sent to the brain whilst the body is in the recumbent position; but the lecturer would rather refer it to a peculiar condition of the encephalic neurine, which favours their production.

The periods of recurrence are very variable; sometimes only a few hours intervene; at others days, weeks, or months.

The causes that produce epilepsy may be various. They may be divided into two classes—*centric*, or those operating from within; and *eccentric*, or those operating from without the nervous centres. The latter are of course less serious in their effects or consequences. Most frequently, perhaps, the paroxysm is the result of eccentric impressions, and may very often be attributed to gastro-intestinal irritation, consequent upon taking indigestible food.

The liability to the disease generally diminishes as age advances, being rarely found to occur after thirty-five years of age. Not unfrequently it disappears during the new evolutions that occur at puberty. The French writers generally divide it into three forms, based upon its intensity, as into *grand mal*, *petit mal*, and *absence*; but as they are all of the same pathological character, and depend on the same causes, the division is scarcely necessary. The attack is frequently preceded by a sense of a vapour or *aura*, which appears to commence at the feet and proceed upwards to the head. This *aura epileptica* is, however, by no means always present.

Sometimes hysterical attacks are mistaken for epilepsy, but in the former there is no loss of consciousness.

Several cases were now brought before the class, as illustrative of the preceding remarks.

*Case first.*—Elizabeth H., æt. 18 years, had been subject to epilepsy, at irregular periods, since she was two weeks old, until last summer, when she experienced an intermission of about three weeks, at which time her catamenia appeared for the first time, and since then they have recurred irregularly. The fits have, however, now become as frequent as ever. This case appears to be hereditary, as her mother was subject to the same affection. The usual remedies, as the different preparations of copper, iron, iodine, &c., have been used without benefit.

*Case second.*—Julia M., æt. 33, unmarried, for the last three years has been subject to fits. The first was induced in her by a severe fright. Not unfrequently—the lecturer remarked—the catamenial discharges are arrested, or rendered very irregular during epilepsy, and, in this instance, the menstruation, which had been regular previously, has been since irregular, although but little inconvenience is experienced from this circumstance.

*Case third.*—Rachael H., aged 33, unmarried, has been subject to the disease ever since her infancy, until within the last two years, when the fits suddenly ceased, since which she has not had one. Has always menstruated regularly, and her general health has been good. The lecturer remarked, that one of the disagreeable results of epilepsy is dementia, or loss of mental power, and such is the case in this woman.

*Case fourth.*—Mary T., aged about 12 years, has had fits ever since she can remember. Her mother was also affected in the same manner. The lecturer has hopes, in this case, that the change which takes place at puberty may arrest the affection.

As in all diseases characterized by paroxysms, little can be accomplished during them except to protect the patient from injuring himself: the great time for action is the interval; yet not much benefit, the Professor thinks, can be derived from antiperiodics. If good is to be expected from any plan, it must be persevered in for a long time.

Antiperiodics are eminently serviceable in diseases which are characterized by paroxysms of regular recurrence; much less so in the same diseases when the intervals are irregular. In epilepsy, the intervals are extremely uncertain, and often very long; so that it is difficult to know how to meet the fits by the ordinary antiperiodics, which are so effective in intermittents. Even where the intermissions are short, the attacks are so irregular as not be treated with ordinary antiperiodics with success. The lecturer remarked, that epilepsy is a disease in which anti-spasmodics, so-called, would seem to be strongly indicated; yet they are rarely employed: and he believed them to be of very limited efficacy. The "direct anti-spasmodics," as assafœtida, castor, dracontium, &c., act only, he conceives, by the new impression which they make on the gustatory nerves, and by their exciting influence on those of the stomach. They could, consequently, only be of service when administered a short time before the paroxysm commences, or during it, which is rarely practicable. The disease generally consists in a pathological state of the organs of innervation, which is best met by remedies that are calculated to give tone to the nervous system, along with attention to diet, taking especial care to avoid any substance that is difficult of digestion. The best tonics are the mineral; and of all remedies which the Professor has employed, he thinks he has derived from nitrate of silver the most benefit. But as the nitrate must undoubtedly become converted into chloride in the stomach, the latter may be given with equal propriety and advantage. Of the nitrate he gives half a gr. three times a day, increasing it by half a gr. a day every fortnight, combined with three or four grains of extract of gentian in the form of pill. Indigo, which was fairly tried in the wards of this Hospital, he conceives to act wholly by the new impression which it makes by quantity on the stomach, when given in very large doses, being in itself inactive; and hence he regards it as no better than so much sawdust. After all, he is disposed to place as much reliance on a strict attention to regimen, and other hygienic measures, as on therapeutical agencies.—Animal food should be preferred to vegetable food, being more easy of digestion. He mentioned, that sometimes an issue, or seton, succeeds apparently in prolonging the intervals; but in the case of Elizabeth H——, it manifestly diminished them, so that the seton had to be withdrawn.

### HYDROPIC CACHEXIA.

A dropsical patient was now introduced, in whom there was infiltration into the cellular tissue of the inferior extremities, and effusion into the peritoneal cavity, as shown by palpation and percussion.

John C——, born in Philadelphia, aged 44, intemperate, a butcher, entered the ward the last day of January, 1844, with general anasarca, which had existed for three weeks previous to his entrance. He had experienced no treatment; he now complains of pain in the umbilical region and legs. The state of the bowels and urine are in all respects natural; the tongue is furred; the sounds of the heart are natural, but somewhat feeble; the abdomen is swollen, and pits on pressure; there is effusion also into its cavity; the urine yields no albuminous deposit when tested with nitric acid; the liver and spleen are of normal size. Has never had jaundice, but had intermittent fever some years ago.

Most of these effusions, the lecturer stated, are symptomatic of the hydropic diathesis, which is characterized, as in this case, by a peculiar paleness of the skin,—*leucophlegmatia*,—but not unfrequently dropsy is connected with diseases or affections of some of the viscera; hence we have *cardiac*, *hepatic*, *splenic*, and *renal* dropsies. In all these cases, there is a manifest want of balance between the absorbents and exhalants. Dropsies, from this fact, may be classified into *active*, where there is an increase in the action of the exhalants, but no diminution of the absorption; and *passive*, when the action of the absorbents is lessened without any increase of the exhalation: in both cases an accumulation of fluid must necessarily result. Sometimes, the accumulation is owing to transudation of the watery parts of the blood through vessels of loose cohesion, owing to some mechanical obstruction to the circulation, either by infarction or induration of a solid viscus, or to disease about the heart or great vessels, which interferes with the general or special circulation. It is always a matter of importance to inquire into these causes. In the case before the class, the dropsy cannot be referred to infarction or induration of the liver or spleen, or to disease of the heart; nor is it a case of renal dropsy; for there is no evidence of disease of the kidney—no *albuminuria*. What then is its nature? The lecturer is disposed to refer it to a general loss of balance between the exhalants and the absorbents of the cellular and serous membranes, induced, perhaps, by irregular habits, for he has been intemperate. The case appears to be as much idiopathic dropsy as any that fall under attention. It is a marked case of hydropic cachexia.

The principal indication is to increase the action of the various emunctories, and to produce a revulsive effect; at the same time to watch closely, and perchance some efficient organic cause of the affection may be discovered.

It was recommended, that a cathartic of jalap and bitartrate of potassa, should be given about twice a week; the latter agent being possessed at the same time of diuretic properties. To assist the diuresis, an infusion of juniper berries with the bitartrate was directed to be taken as an ordinary drink when the patient was thirsty. A grain of the mild chloride of mercury, with a grain of squill, was also prescribed night and morning, to be continued so as to touch the mouth slightly. In this way the revulsive effect of the mercury will be secured, together with the diuretic action of the squill. The Professor expressed his hopes, that as there was no manifest visceral disease, under the combined agency of these remedies, with a well regulated diet, the effused fluid would be absorbed, and the patient ultimately recover, provided his stamina be good, and he be careful, by regularity in his habits, not to endanger a recurrence.

